# In Silico Molecular Modeling of Four New Afatinib Derived Molecules Targeting the Inhibition of the Mutated Form of BCR-ABL T315I

**DOI:** 10.3390/molecules29174254

**Published:** 2024-09-08

**Authors:** Kelvyn M. L. Rocha, Érica C. M. Nascimento, Rafael C. C. de Jesus, João B. L. Martins

**Affiliations:** 1 Department of Pharmacy, Faculty of Health Sciences, University of Brasília, Brasília 70910-900, DF, Brazil; kelvynmagalhaeslr@gmail.com (K.M.L.R.); ericamoreno@unb.br (É.C.M.N.); rafaelcamposunb@gmail.com (R.C.C.d.J.); 2 Computational Chemistry Laboratory, Institute of Chemistry, University of Brasília, Brasília 70910-900, DF, Brazil

**Keywords:** CML, TKI, BCR-ABL, molecular modeling, NCI

## Abstract

Four afatinib derivatives were designed and modeled. These derivatives were compared to the known tyrosine-kinase inhibitors in treating Chronic Myeloid Leukemia, i.e., imatinib and ponatinib. The molecules were evaluated through computational methods, including docking studies, the non-covalent interaction index, Electron Localization and Fukui Functions, in silico ADMET analysis, QTAIM, and Heat Map analysis. The AFA(IV) candidate significantly increases the score value compared to afatinib. Furthermore, AFA(IV) was shown to be relatively similar to the ponatinib profile when evaluating a range of molecular descriptors. The addition of a methylpiperazine ring seems to be well distributed in the structure of afatinib when targeting the BCR-ABL enzyme, providing an important hydrogen bond interaction with the Asp381 residue of the DFG-switch of BCR-ABL active site residue and the AFA(IV) new chemical entities. Finally, in silico toxicity predictions show a favorable index, with some molecules presenting the loss of the irritant properties associated with afatinib in theoretical predictions.

## 1. Introduction

Myeloid leukemia is a neoplasm characterized by the clonal proliferation of myeloid leukocytes [1]. These cells typically present a primitive morphology, also known as left-shift, when viewed in a blood smear due to their early release to the bloodstream [2]. Chronic myeloid leukemia (CML), despite not presenting significant symptoms, with only splenomegaly [3,4] and a slightly altered blood smear as the most described signals of the disease [4], can lead to a delayed diagnosis that favors the disease evolution [5,6,7,8,9].

CML is known for being one of the first diseases whose molecular mechanism was identified, rendering it a status of model disease. It is known that CML has very close ties with the expression of the Philadelphia (Ph) chromosome [10,11,12]. The Ph chromosome is a result of the translocation involving the chromosome pairs 9 and 22 [13], which yields a chimeric assembly that can, through translation mechanisms, produce the BCR-ABL enzyme [14,15,16].

**Figure 1:**
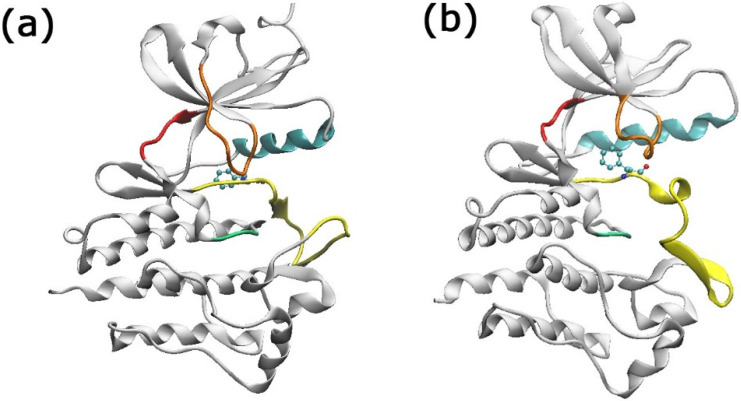
The BCR-ABL enzyme in its (**a**) DFG-in (PDB: 2GQG) and (**b**) DFG-out (PDB: 3QRJ) conformations. Color code: P-loop orange, c-alpha helix cyan, hinge red, catalytic loop green, and activation loop yellow. The Phe382 residue is shown in the CPK representation.

The tyrosine kinase enzyme BCR-ABL [14,15,17] is crucial for the oncological development of CML patients. It is responsible for producing constant proliferation signals [18,19]. Structurally, BCR-ABL presents several important sites, such as the gatekeeper residue (located at the Ile/Thr315 region), the DFG switch (Glu381, Phe382, and Gly383), the P-loop (Met344, Gly250, Tyr254, and Glu255), and the Met318 resid [20,21,22]. The DFG switch site (Figure 1) determines the activity of the protein through its conformations: “in” and “out” [20]. The relevance of the BCR-ABL enzyme as a critical target for treating CML patients underscores the recent studies of this enzyme [13,15,16,17,23,24].

The treatment of this disease has posed a challenge since the discovery of the Ph chromosome [25]. Imatinib was the first successful molecule that emerged from a rational drug design used for CML treatment [26,27]. The second generation of tyrosine kinase inhibitors (TKI) improved over imatinib resistance but could not overcome the T315I mutation [28,29,30,31]. The therapeutic resistance in CML treatment provided by the T315I mutation would be tackled only in the third generation of molecules. Ponatinib is a third-generation TKI developed through rational design targeting the highly resistant forms of the BCR-ABL kinase [32,33], inhibiting the T315I BCR-ABL isoform. The molecular structure of ponatinib provides further insight into the inhibitory activity of the TKI molecule. Although ponatinib proved to be an invaluable alternative, it is also associated with severe cardiovascular side effects and ischemic strokes [22,34].

Afatinib (AFA) is a TKI molecule associated primarily with treating lung cancer. It has proven versatile and presents the potential for also treating breast and colon cancers [35,36,37,38,39]. This molecule has been studied in the context of myeloid leukemia, revealing modest results when applied as it is but demonstrating that some structural modifications in its molecule contribute to enhancing the inhibitory activity [40]. Some new quinazoline and pyrimidine derivatives have been studied in the context of repurposing drugs [41,42,43,44,45].

Understanding electronic structure is paramount in drug design, as it provides insights to elucidate potential interaction sites with biological targets at the molecular level [23,40,42,46,47,48,49]. It can aid in predicting ligand–receptor binding affinity, intensity, nature of interactions, and molecular reactivity. The electronic structure study sheds light on critical regions of the derived molecules and the established inhibitors. Additionally, further information may be obtained by evaluating the molecular descriptors and the potential of the derived molecules as new drugs. One can derive the importance of these properties since computer-assisted electronic studies and analysis of molecular descriptors have provided significant benefits in modeling new drugs [50].

**Figure 2:**
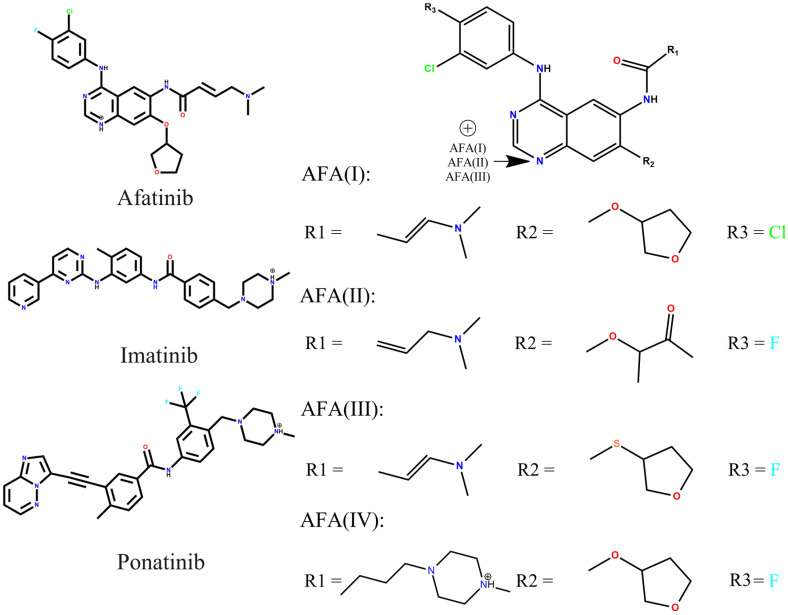
Representation of the 2D molecular structures of afatinib, imatinib, and ponatinib, and the afatinib derivatives, AFA(I), AFA(II), AFA(III), and AFA(IV).

In this context, considering the need for efficient molecules with potential for BCR-ABL inhibition, we have studied four molecular structures derived from afatinib and compared them to imatinib and ponatinib (Figure 2). The aim is to describe the main interactions and electronic properties that account for the better score of these molecules. Electronic structure study at the Density Functional Theory (DFT) level and molecular docking was used to predict ligand–receptor binding affinity, frontier molecular orbitals, nature of interactions, and molecular reactivity.

## 2. Results and Discussion

### 2.1. Electronic Structure, Frontier Molecular Orbitals

**Figure 3:**
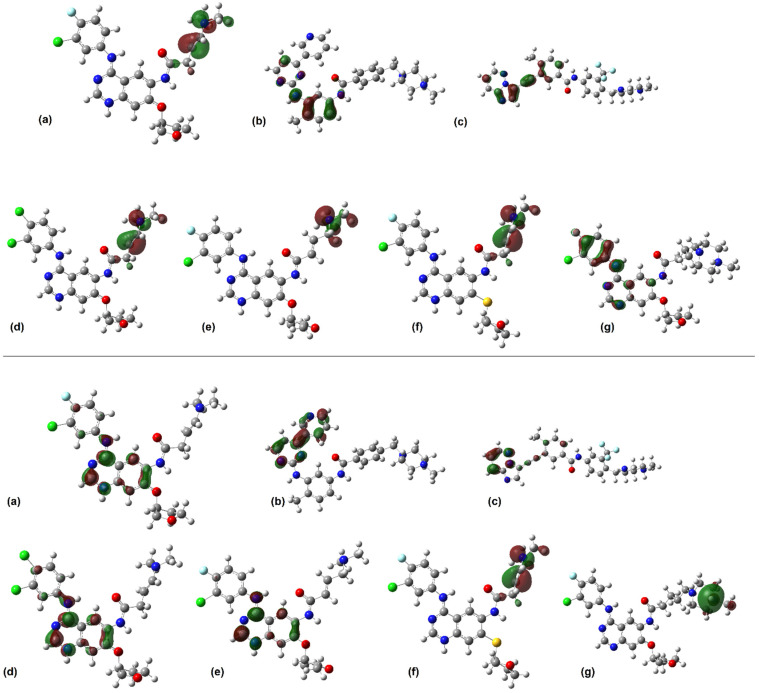
Isosurfaces of the frontier orbitals of (**a**) afatinib, (**b**) imatinib, (**c**) ponatinib, (**d**) AFA(I), (**e**) AFA(II), (**f**) AFA(III), and (**g**) AFA(IV) at B3LYP/6-311+G(d,p) level. The HOMO isosurfaces are depicted at the **top**, and the LUMO isosurfaces at the **bottom**. The images were rendered with an isovalue of 0.05 e-.Å^−3^.

The distribution and localization of the HOMO (Highest Occupied Molecular Orbital) and LUMO (Lowest Unoccupied Molecular Orbital) of the calculated ligands are depicted in Figure 3.

Three of the studied molecules (Figure 3d–f) presented similar molecular orbital distributions when compared between themselves and the afatinib molecule (Figure 3a). The HOMO orbital is distributed over the scaffold of the 4-dimethylamino-but-2-enamide group around the tertiary amine in the AFA(I), AFA(II), and AFA(III) structures. On the other hand, AFA(IV) presents a HOMO distribution resembling the one shown by ponatinib, with concentration over aromatic centers. Since the HOMO isosurfaces can indicate sites prone to intermolecular interactions, the modeling of AFA(IV) was successful in resembling this aspect of ponatinib. It may facilitate the establishment of effective intermolecular interactions with the target enzyme.

The LUMO sites may also indicate interaction-prone scaffolds. The LUMO distributions occupy aromatic centers, the 3-chloro-4-fluoroanilino and quinazoline groups in most molecules. AFA(IV) differs from all others, having a LUMO distribution centered over the methylpiperazine moiety, which may prove to be a hindrance to the performance of this ligand. Most of the derived molecules were able to mimic the LUMO distributions of afatinib, which may prove valuable when considering their interaction with the BCR-ABL active site residues.

### 2.2. Fukui Functions

Fukui functions [51] are essential in the field of theoretical chemistry for their usefulness in predicting reactivity based on principles of the molecular orbital theory. There are three main functions, and they are described as follows:

1. *f*^0^: This function describes the tendency of electrons to flow in and out of a specific region in the molecular structure. This function can be used to indicate regions associated with radical attacks in molecular entities.
2. *f*^+^: This function represents nucleophilic attack potential. Regions within a molecule that concentrate this function are where electrons are most likely to be donated, indicating high reactivity towards nucleophiles.
3. *f*^−^: This function represents the electrophilic attack susceptibility, identifying the moieties where electrons are most likely to be accepted.

**Figure 4:**
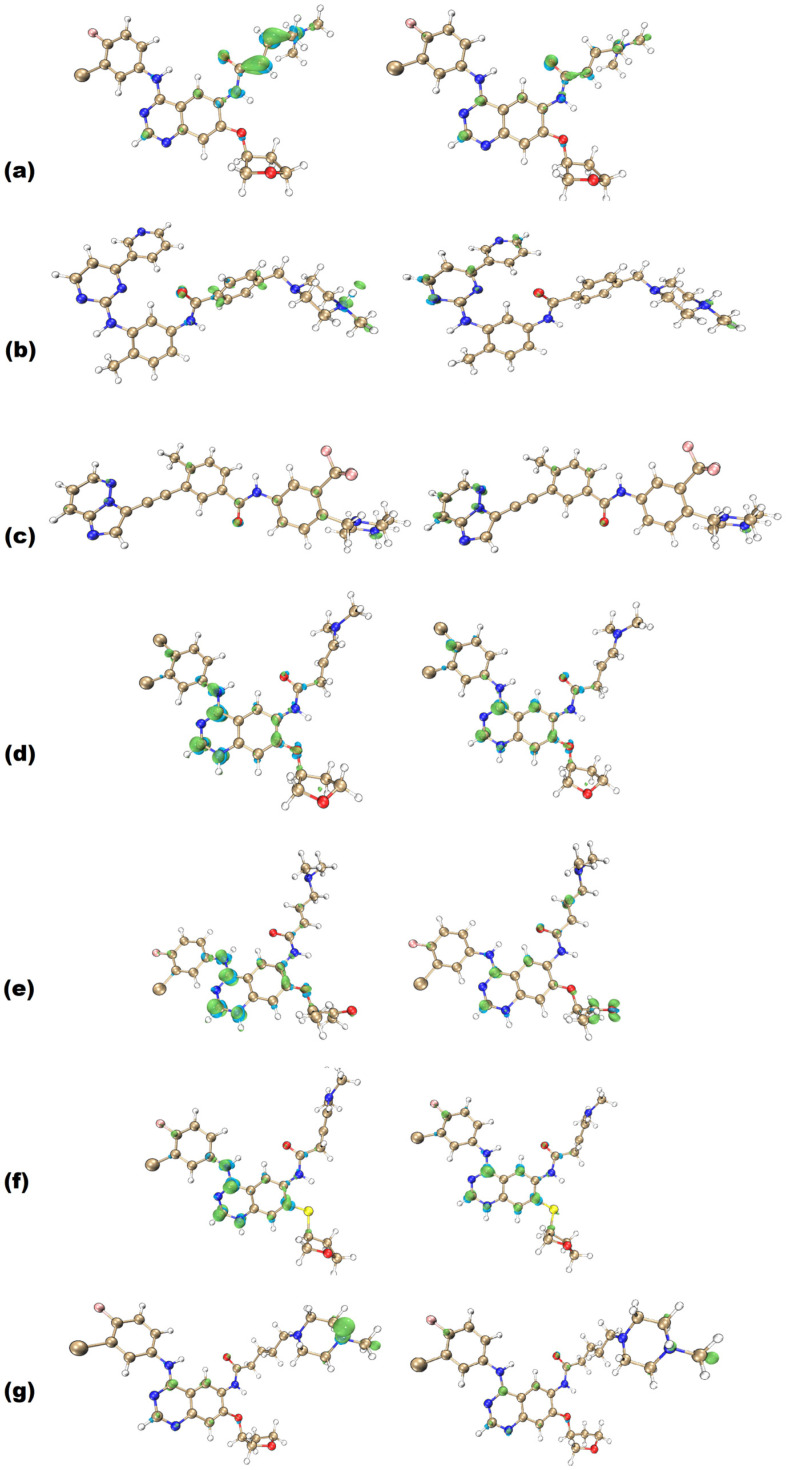
Fukui functions (*f*^−^ at the left, *f*^+^ at the right) of (**a**) afatinib, (**b**) imatinib, (**c**) ponatinib, (**d**) AFA(I), (**e**) AFA(II), (**f**) AFA(III), and (**g**) AFA(IV) at B3LYP/6-311+G(d,p) level. Positive regions are represented in green, while negative regions are presented in cyan. Rendering was obtained with an isovalue of 0.005.

The Fukui functions of all molecules are presented in Figure 4. Those functions provide some insights into the nature of the studied BCR-ABL inhibitors and afatinib derivatives.

Firstly, it is possible to notice that most of the established classical TKI molecules have *f^−^* as their most prominent function. This feature can also be observed in ponatinib, albeit to a lesser degree, with some additional *f^−^* sites over the trifluoromethyl aromatic ring. Some *f*^−^ sites also coincide with the *f+* distributions. The prominence of *f^−^* can be one of the beneficial aspects for competitive inhibition of BCR-ABL since it resembles the profile of ATP [52]. When evaluating the Fukui functions of ATP structure (Figure S1, Supplementary Material), it is possible to identify that the *f^−^* isosurface also has a larger density than *f^+^.* The studied molecules also show a more prominent *f^−^* region that is equally present in imatinib and ponatinib. This trend is a promising indication that the studied ligands may be able to effectively compete with the ATP structure due to their similarities in the *f^−^* function distribution and intensity.

Also, ponatinib shows coinciding *f^+^* and *f^−^* regions like the AFA-derived molecules do. On the other hand, imatinib does not show this behavior, indicating that the coinciding Fukui functions may be desirable for inhibiting the imatinib-resistant BCR-ABL enzyme. If this suggestion is correct, the derived molecules appropriately approached ponatinib in this regard and may be associated with better inhibition performance.

The Fukui functions also indicate probable sites for establishing intermolecular interactions. The *f^−^* functions over the polar tail in afatinib molecule, the methylpiperazine ring amide group in imatinib, the aromatic rings in ponatinib, the aromatic centers and amide groups in AFA(I) and AFA(III), the carbonyl group in AFA(II), and the methylpiperazine ring in AFA(IV) all indicate that these regions can perform important roles when interacting with the target protein.

The *f^+^* function seems to roughly coincide in all molecules, and they tend to be close to the *f^−^* function or even located at the same sites. This means these regions are versatile regarding intermolecular interactions and can be recognized by most amino acid chains.

The central amide moiety of ligands plays an important role in inhibiting the BCR-ABL protein [33,53]. It is one of the regions where a hydrogen bond must be established between the ligand and the DFG residues Asp381 and Phe382, effectively taming the DFG switch [33,53]. The afatinib, imatinib, and ponatinib molecules show that this region concentrates mainly on the *f^−^* function. The derived molecules also concentrate on the *f^−^* function. The results provided by the Electron Localization Function analysis around this region are analyzed to better understand the central amide and its vicinity.

### 2.3. Electron Localization Function Analysis

Non-covalent interaction analysis is crucial for understanding the ligand and ligand–protein interactions to account for the weak interactions. Non-covalent interactions (NCI) index, Electron Localization Function (ELF), and atoms in molecules (AIM) have been widely used to study TKIs [46,54,55,56,57,58,59,60]. The ELF is a powerful tool for understanding the chemical and electronic nature of intramolecular and intermolecular interactions [61]. The ELF method is a quantum chemical approach that can be applied to biological ligands to allow the visualization and analysis of chemical bonding and electron density distributions, which are fundamental characteristics in the context of drug-receptor interactions.

**Figure 5:**
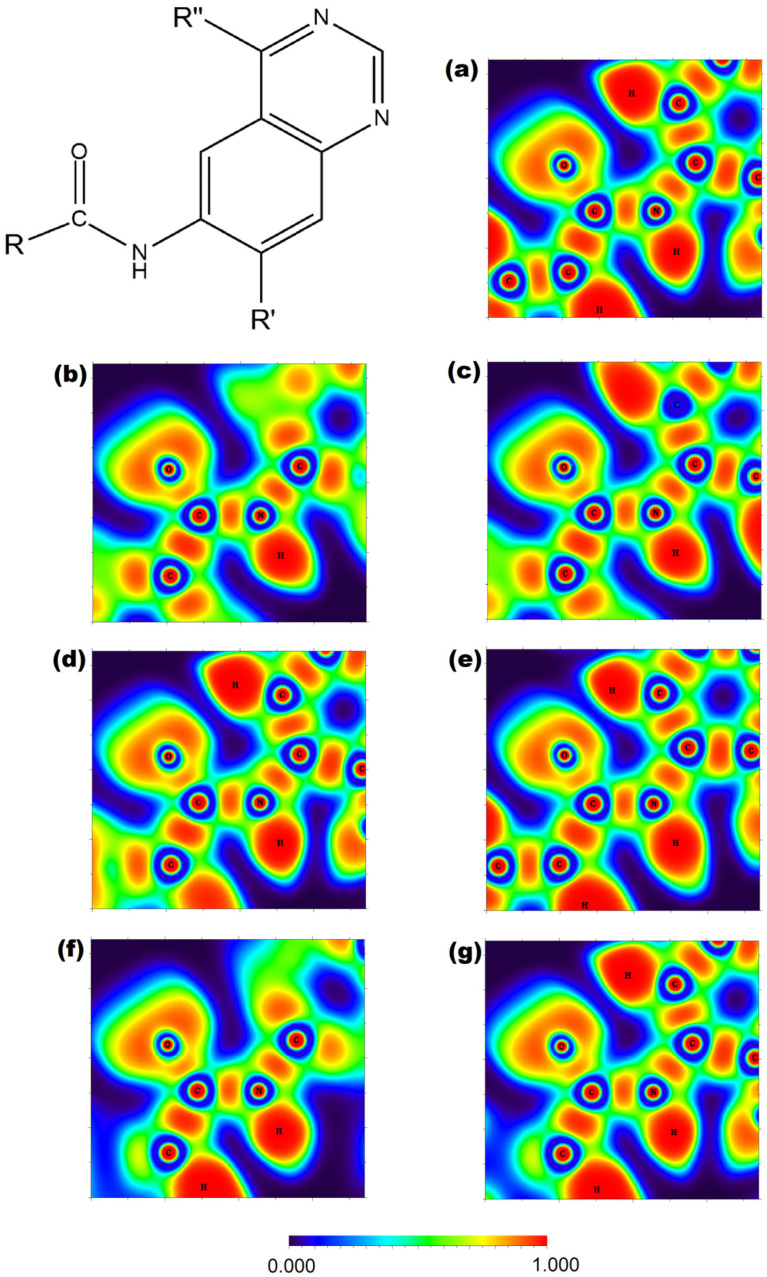
Plotting of the 2D-Electron Localization Function around the central amide moiety at B3LYP/6-311+G(d,p) level. The scaffold represents the afatinib derivatives. (**a**) afatinib, (**b**) imatinib, (**c**) ponatinib, (**d**) AFA(I), (**e**) AFA(II), (**f**) AFA(III), and (**g**) AFA(IV).

The use of the ELF method has allowed for mapping the electron density (Figure 5) of the ligands studied in this work, which is valuable in providing some insight into the behavior of the amide moiety present for the effective inhibition of the BCR-ABL protein.

When evaluating the ELF plotting of ponatinib, it is possible to notice that the aromatic ring adjacent to the amide group concentrates high electronic density over its hydrogens in most molecules, with imatinib showing slightly less negative hydrogens. Ponatinib has carbon atoms with low electron densities according to the scale, and upon joint inspection of Figure 2 and Figure 5, one can safely assume the R-CF_3_ scaffold causes this due to its inductive effect [49]. This effect may also be responsible for intensifying the electronic densities over neighboring hydrogens, which may be related to the inhibitory profile of ponatinib. It was possible to observe that afatinib also has a hydrogen atom with these described properties. Still, it differs from ponatinib since different distributions can be observed over the start of the carbon side chain. However, the derived molecules were able to approach ponatinib in this regard. Since all molecules showed intensely populated atoms in this region, the amide moiety is a prime site for intermolecular interactions and pharmacological recognition [54,55,62].

Table 1 shows the bond critical point properties of the amide group. Imatinib values of ρ and $\nabla^{2}\rho$ are out of the range of afatinib and ponatinib. In general, AFA(III) and AFA(IV) have the same behavior as ponatinib, with AFA(IV) having greater similarity with ponatinib using the critical points of Quantum theory of Atoms in Molecules (QTAIM) [63].

Imatinib does not have high electronic densities over the hydrogens depicted in Figure 5, which may explain its lesser effectiveness when applied to the mutated protein. This result can be confirmed upon inspecting the Fukui functions of imatinib in Figure 4, where no isosurface is observed over the aromatic ring adjacent to the nitrogen of the amide group. This profile may be associated with improper interactions that hinder the effectiveness of the imatinib molecule inhibiting the mutated protein.

On the other hand, the derived molecules were able to maintain high electronic density over the aromatic ring hydrogens, presenting a similar profile as the ponatinib molecule. This electronic density also corroborates what is shown in Figure 4, which shows that at least one of the represented isosurfaces is concentrated over the aforementioned aromatic ring. The *f^−^* isosurface of ponatinib coincides with the intense electronic densities shown in Figure 5, which indicates that this moiety may be an important element inhibiting the target enzyme.

### 2.4. ADMET Evaluation

Absorption, Distribution, Metabolism, Excretion, and Toxicity (ADMET) studies are crucial in drug discovery and development and are fundamental to ensure the safety of human use of derived molecules, being applied in a range of fields in drug discovery [55,64,65].

A valuable initial filter during the early stages of drug development, such as in computational studies, is the set of guidelines known as Lipinski’s Rule of Five (Ro5) [66,67,68]. These are empirical instructions based on physicochemical properties that allow us to assess the likelihood of a molecule’s effectiveness as an orally administered drug. The rules are defined as follows: (i) The molecular weight should be less than 500 Da; (ii) The partition coefficient (LogP) should be less than 5; (iii) The number of hydrogen bond acceptors (#H-acc) should be less than 10; and (iv) The number of hydrogen bond donors (#H-don) should be less than 5.

To further evaluate the behavior of the studied molecules regarding the Ro5, we present the data obtained through the computational tools SwissADME [69] and Osiris [70] in Table 2.

As shown in the values of Table 2, the molecules follow the Ro5 with a maximum of one violation; the molecular weight was larger than 500 Da in all violating structures: AFA(I), AFA(III), and AFA(IV). That violation is the same as that presented by ponatinib, the most effective inhibitor against T315I BCR-ABL. It indicates that a smaller molecular weight is not critical for its pharmacokinetic and inhibitory properties.

All derived molecules presented a partition coefficient smaller than five, which is recommended by the Ro5 and expected from molecules applied to the BCR-ABL enzyme since most figures described for CML drugs are about four [71], which is corroborated by our simulations. Therefore, the derived molecules of afatinib have an adequate partition coefficient in the membrane permeability, which is needed to be used as a potential drug, in this case, for the inhibition of the BCR-ABL enzyme. Table 3 shows the ADMET data of the studied molecules. 

According to the results of the toxicity evaluation (Table 3), all derived molecules maintain an acceptable predicted safety level. Particularly, when we consider AFA(II) and AFA(IV), both present no predicted risk in all the evaluated possibilities; within the estimate, this data possibly poses an improvement over afatinib since the modifications may be effective in eliminating its irritating properties, which are described in previous works [72,73].

### 2.5. Molecular Docking Studies

Docking studies are computational methods used in the drug discovery field to predict affinities and binding conformations of ligands regarding the target proteins. These studies play crucial roles in early development, providing insight into important interactions involving the studied drugs. With computer hardware and software advances, docking studies have become cornerstones of drug development, indicating optimal strategies and avoiding lengthy experimental testing.

To further understand the behavior of the molecular structure entities, we performed docking and NCI studies to gain insights into the potential inhibitory effect of the derived molecules and compare each effect with the classic TKI molecules. The binding energy values of the interactions (scores) are analyzed to evaluate the general trend applied to the BCR-ABL protein and are presented in Table 4. 

The studied molecules can be divided into four regions, as shown in Figure 5: the R1, R2, and R3 groups and the central aromatic rings. Upon analyzing the scores obtained through the docking studies, the modifications improved the score compared to afatinib. The most effective modification was the addition of a methylpiperazine ring R1 group of afatinib molecule, generating the AFA(IV) candidate, which provided a score of −9.0 kcal/mol against the imatinib-resistant protein, further suggesting the importance of the methylpiperazine moiety. None of the modifications were effective against the imatinib-susceptible BCR-ABL isoform. Although an improvement could be identified compared to afatinib, none of the derived molecules posed a significant upgrade over ponatinib or imatinib. Nevertheless, considering the improving results when compared to afatinib, there is an indication that the modifications, especially in the case of the AFA(IV), pose a first step into the development of a new derivative with the potential to inhibit the BCR–ABL enzyme. This reinforces the versatility of afatinib as a starting point for the design of derived molecules that can be effective against other receptors as well as its original target [76].

**Figure 6:**
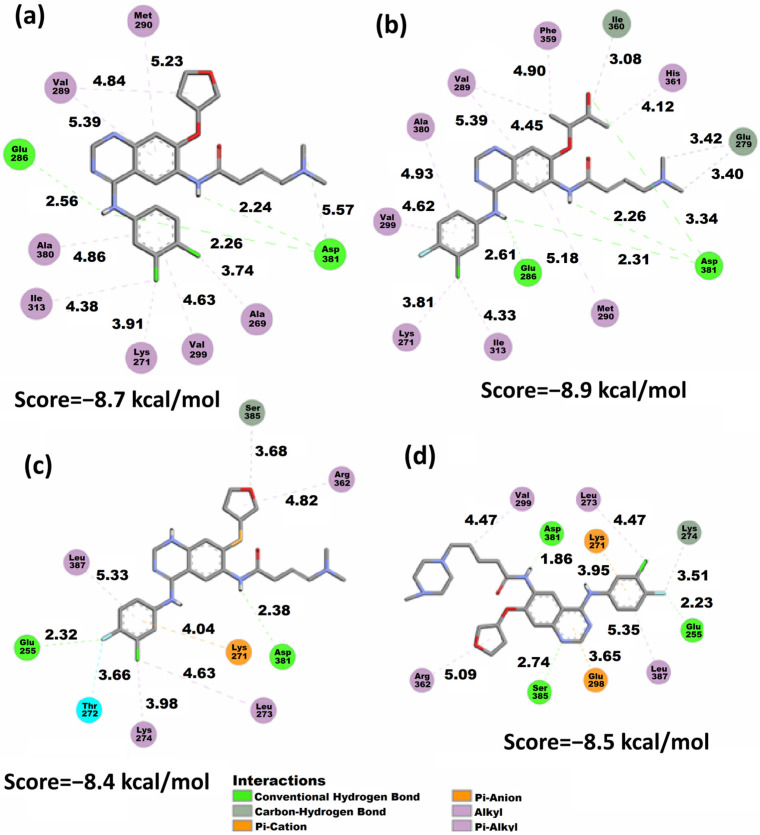
2D representation of the Vina docking conformations in the 1OPJ protein showing the distances (Å) of calculated structures. (**a**) AFA(I), (**b**) AFA(II), (**c**) AFA(III), and (**d**) AFA(IV).

The results obtained with the Vina algorithm tended to associate better scores in the case of the AFA(IV) and AFA(III) ligands in the mutated isoform of the enzyme (3QRJ). This indicates that the sulfur atom substitution also proved to be well-received in the potential inhibition of the mutated protein [15]. Additionally, the conformations associated with each Vina score are presented in Figure 6. The classical inhibitors are presented in the supplementary material (Figure S2).

The docking conformations presented in Figure 6 and in the supplementary material showed the behavior of all the studied molecules when docked to the wild-type protein. It is possible to observe that AFA(II) is the best-score molecule, establishing three strong hydrogen bonds with the Asp381 residue involving the amine and carbonyl of the amide group, which suggests the high stability achieved by the protein and AFA(II) complex. The carbonyl and amine groups were also shown to be populated sites regarding the distribution of the *f^−^* isosurface (Figure 4) in AFA(II) and to a lesser degree in AFA(I), AFA(III) and AFA(IV), indicating that this property affects better recognition of the DFG-switch through the Asp381 residue. This hypothesis is reinforced by imatinib, a ligand that presents one strong hydrogen bond formed between Asp381 and the oxygen of the amide group belonging to imatinib, which also concentrates an intense *f*^−^ isosurface density.

**Figure 7:**
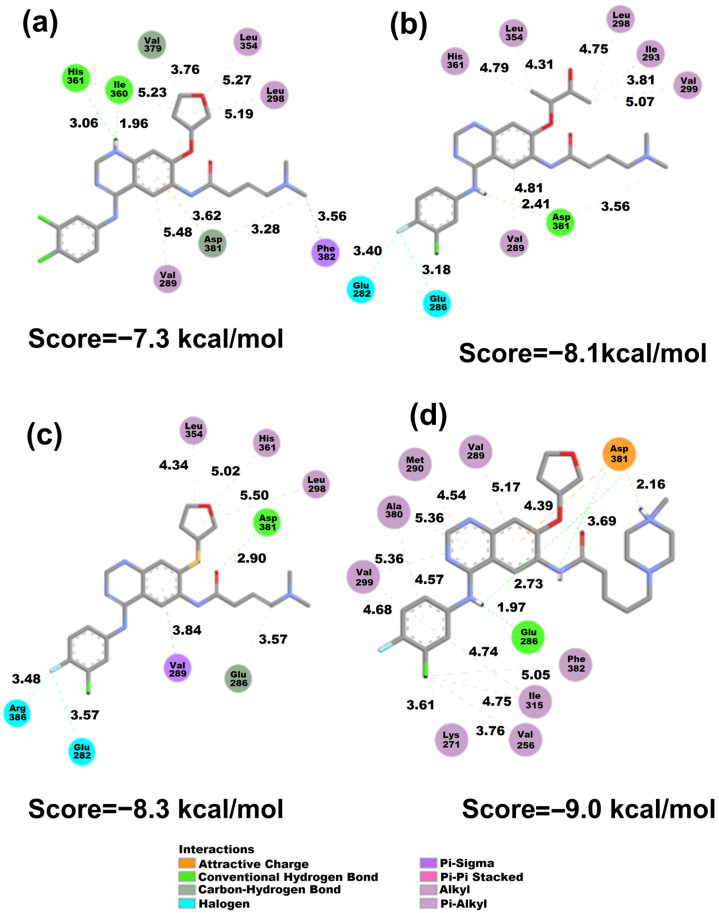
2D representation of the Vina docking conformations in the 3QRJ protein. Distances are measured in Å. (**a**) AFA(I), (**b**) AFA(II), (**c**) AFA(III), and (**d**) AFA(IV).

Considering that Vina provides much accuracy for highly flexible molecules [77], we can infer that although the addition of the methylpiperazine ring is beneficial, some other characteristics (such as volume or high flexibility) may be hindering the inhibitory potential of AFA(IV) when applied to the wild-type enzyme. Figure 7 shows the docking conformations achieved by all the derived ligands in the mutated protein. The classical inhibitors are presented in the supplementary material (Figure S3).

Although both AFA(II) and AFA(IV) obtained moderate improvement in docking scores when applied to the resistant enzyme, AFA(IV) was only able to approach the score of imatinib. Thus, AFA(II) and AFA(IV) still cannot be labeled as effective inhibitors, but the strategies applied in their modeling can be put forth as a good starting point. The interaction of the methylpiperazine ring and Asp381 was present in AFA(IV), and it is deemed as a key interaction for the inhibition of BCR-ABL.

When evaluating AFA(IV) and its docking representation (Figure 7), several interactions established with the Asp381 residue through hydrogen bonds and attractive charges can be seen at a very close distance (3.69 and 2.16 Å). Additionally, a hydrogen bond (1.97 Å) with the Glu286 residue and a π-Alkyl interaction with the mutated gatekeeper residue generates a similar behavior to what is present in ponatinib and imatinib. 

According to the docking study, AFA(IV) has no significant difference from imatinib, with only 0.1 kcal/mol of interaction energy difference, and it failed to achieve a score at the same level as ponatinib. However, AFA(IV) achieved the purpose of this study, which was to improve the score and interactions of afatinib for the mutated protein. This low score may be explained by the absence of interaction with the Met318 residue, deemed important for effective inhibition [22]. As shown in the wild-type protein, meaningful interactions tend to be established in regions with some degree of *f^−^* isosurface population, with some examples being the amine and amide groups in AFA(I), the amine and carbonyl groups in AFA(II), the amide group in AFA(III), and the methylpiperazine ring in AFA(IV). Although AFA(IV) established a hydrogen bond with the Asp381 residue, AFA(IV) interacted as an H-bond donor instead of an acceptor as in ponatinib. This may be contributing to the lower scores of the derived molecules.

LUMO is in the methylpiperazine ring (Figure 3) of AFA(IV), and *f^−^* is also present in the AFA(IV) and ponatinib methylpiperazine ring. Furthermore, a hydrophobic pocket of residues interacts with the fluoro-chlorine ring, and one of the residues is Ile315. These interactions are probably affecting the AFA(IV) score.

Considering the score values shown in Table 4 while also evaluating the interactions shown in Figure 7, the behavior of the ligands regarding the Arg386 and Met318 residues was hypothesized to be modulating the effectiveness of the docking conformation. Arg386 is involved in the regulation of the DFG-in and DFG-out conformations, being an important target for the strategy of conformational control [78]. However, in the context of competitive inhibition, the affinity for the Arg386 residue seems to be disruptive in nature, being present in the worst-score docking models as seen in AFA(III) applied to the mutated protein. The proximity and interaction with the Arg386 residue may compete with other residues, such as the DFG-switch and Met318, which are located deeper into the binding pocket. To test this hypothesis, the tridimensional representations of the docking models were analyzed through an NCI study, and the distances between the ligands and key residues were also monitored.

### 2.6. Non-Covalent Interactions Index

NCI refers to the attractive or repulsive forces between molecules that do not involve the sharing of electrons, such as hydrogen bonds, van der Waals forces, and electrostatic interactions [79,80]. These interactions are critical for many biological processes, including protein folding, DNA replication, and enzyme catalysis.

**Figure 8:**
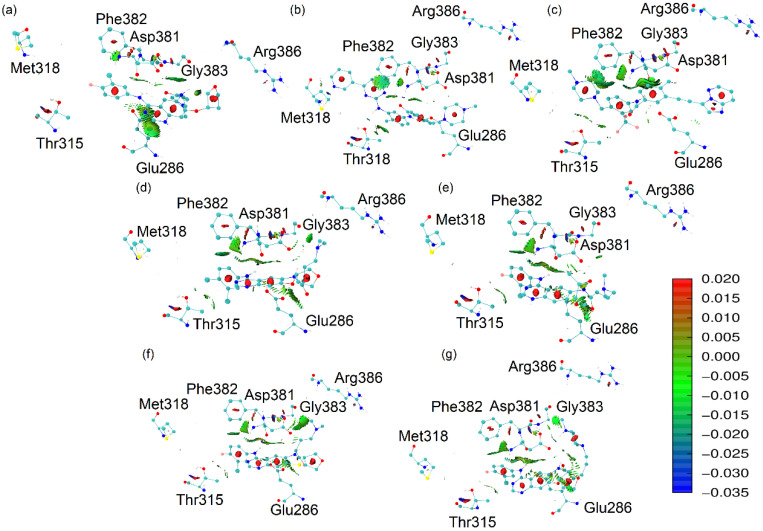
NCI analysis based on promolecular densities of the docked conformations of the ligands in the 1OPJ protein. (**a**) afatinib, (**b**) imatinib, (**c**) ponatinib, (**d**) AFA(I), (**e**) AFA(II), (**f**) AFA(III), and (**g**) AFA(IV).

For their critical role in defining drug effectiveness, the understanding offered by an NCI study is significant for designing new drugs and elucidating the behavior of target proteins, ligands, and protein–ligand complexes. Figure 8 presents the NCI isosurfaces of the docked complexes with the 1OPJ wild form. We have used the promolecular electronic densities, where the electronic density of the system is evaluated based only on the structure and superposing the built-in atomic densities in their free states [79]. The color map is coded by attractive (blue), repulsive (red), and van de Waals (green) interactions.

Upon observing the interactions established between the ligands and the 1OPJ wild form of the target protein, the interactions between the Thr315 residue and the best-scoring ligands in Figure 8 (ponatinib and imatinib) are very clear. They consist of an intensely attractive hydrogen bond involving imatinib (3.17 Å) and a van der Waals interaction involving ponatinib (3.74 Å). The affinity for the Thr315 sidechain appears to be proportional to the effectiveness of the inhibition.

Imatinib also presented a significant interaction with the Met318 residue. It is a hydrogen bond involving one of the pyridine rings of imatinib and the hydrogen donor of the Met318 backbone. This interaction is possibly related to an effective docking model [22]. It might be the reason for the better score of imatinib when compared to ponatinib in the context of the imatinib-susceptible enzyme.

Afatinib did not show significant interactions with either the Thr315 or the Met318 residues, which is probably related to its poor score when docked to the oncoprotein [22]. On the other hand, afatinib shows some attractive interactions with the DFG-switch, which is denoted by the green shades and indicates that although afatinib is not able to reach an optimal position for the inhibition of the target enzyme, it can approach the DFG-switch.

**Figure 9:**
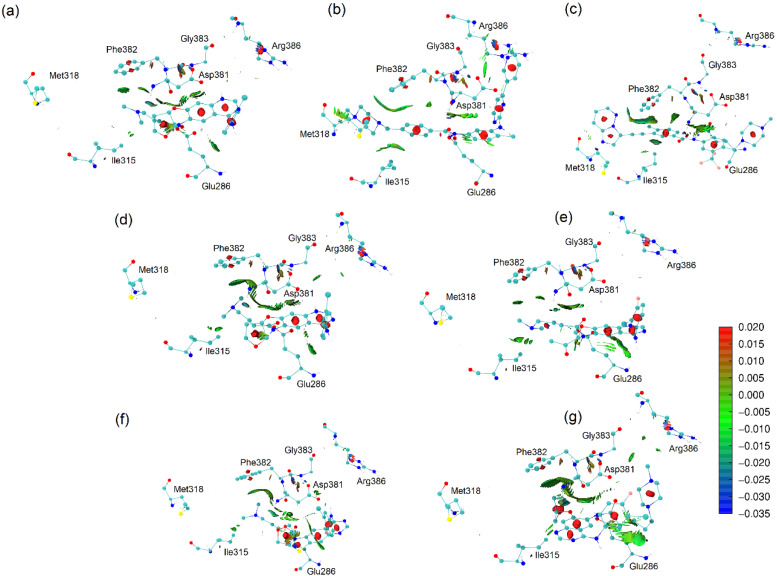
NCI analysis based on promolecular densities of the docked conformations of the ligands in the 3QRJ protein. (**a**) afatinib, (**b**) imatinib, (**c**) ponatinib, (**d**) AFA(I), (**e**) AFA(II), (**f**) AFA(III), and (**g**) AFA(IV).

As hypothesized, the Arg386 proximity is indeed present only in the worst-score molecules in complex with the wild form of BCR-ABL enzyme: AFA(III) (4.81 Å) and AFA(IV) (4.98 Å). Therefore, this residue may disrupt the interactions that should be established with the ligand for effective competitive inhibition of the catalytic activity. Instead, residues that effectively interact with this residue may present potential for the alternative strategy of conformational control. Figure 9 shows the NCI analysis of the ligands in the 3QRJ protein.

The classical TKI and best-score molecule, ponatinib, presented significant interactions with residues Ile315 and Met318. Afatinib did not interact significantly with the Met318 residue and interacted lightly with the Arg386 sidechain. Thus, when cross-referenced with the score of afatinib, it poses another piece of evidence suggesting that the affinity for the Arg386 may be responsible for hindering the competitive inhibitory potential of TKI molecules in the BCR-ABL enzyme.

Imatinib interacts well with the Met318 and Ile315. Still, it also presents an intense van der Waals interaction with the Arg386 residue, which might be the cause for the less favorable score of this ligand when compared to ponatinib, as explained further. AFA(IV), the best-score derivative of afatinib for mutant BCR-ABL (PDB 3QRJ structure), placed its methylpiperazine ring in the vicinity of the DFG-switch, allowing for the attractive charge interaction with the Asp381 residue as well as a hydrogen bond between the amide group of the ligand and the backbone of Asp381, in a similar fashion to ponatinib [33]. These results suggest that further modifications of the molecule might be beneficial and that adding a methylpiperazine ring proved to be a suitable first modification.

**Figure 10:**
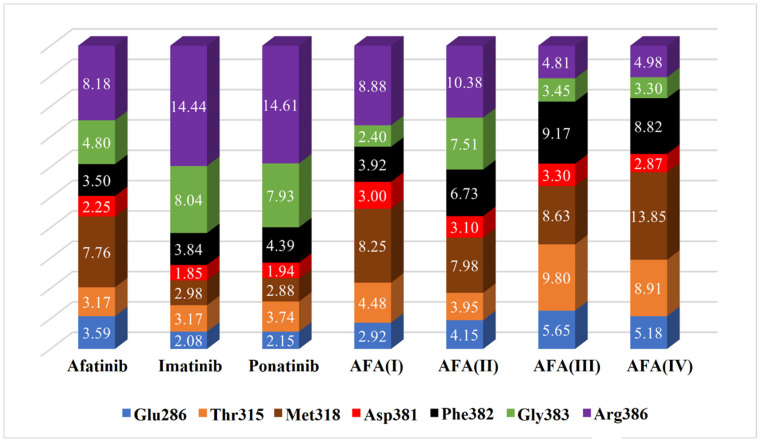
Estimated distances (Å) between key residues and each ligand in the chosen docking conformations for the wild-type enzyme (PDB 1OPJ). The volume of each bar is normalized.

**Figure 11:**
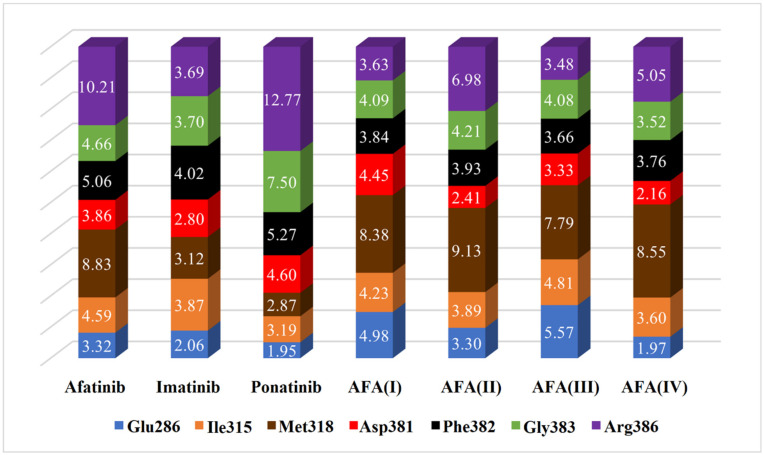
Estimated distances (Å) between key residues and each ligand in the chosen docking conformations for the mutated enzyme (PDB 3QRJ). The volume of each bar is normalized.

To better understand the deviations presented by the scores provided by the docking simulations, the profile of some important interactions was thoroughly observed. Specifically, we have calculated the smallest distances between the atoms of the ligands and the closest atoms of the key residues Thr/Ile315, Met318, Arg386, Asp381, Phe382, Gly383, and Glu286, as shown in Figure 10 and Figure 11. The stacked bar graph values are normalized and refer to the smallest distance. These residues are frequently involved in important interactions involving the target enzyme and all ligands, possibly modulating the energetic effectiveness of the complex.

Comparing the estimated distances and scores obtained in the docking studies, it is possible to note that the interaction with the Arg386 residue must be avoided in both enzyme isoforms, as well as the Gly383 to a lesser degree, since these residues may be able to disrupt the optimal conformation and inhibitory interactions resulting in the hindering of the effective positioning of the ligand. That can be observed in AFA(III) and AFA(IV) for the wild-type protein since they are the worst-score molecules and present small distances to the residues above. 

The distances between the cited residues of BCR-ABL and the TKIs ponatinib and imatinib suggest that the residues Thr/Ile315, Met318 (especially in the mutated enzyme), and Glu381 must be the targets of closer interaction. It is possible to observe that AFA(II) and AFA(IV) can mimic the distances showed by ponatinib to some degree in the mutated enzyme, which explains the better scores associated with them. According to score values described in Table 4, AFA(IV) was able to surpass imatinib by 0.1 kcal/mol when applied to the resistant enzyme according to the Vina docking algorithm, indicating high affinity regarding the BCR-ABL protein was achieved through our modification strategy. Since this was only observed in the mutated variant of the enzyme, it is possible to infer that AFA(IV) is more adequate for resistant forms of the protein, while AFA(II) proved to be more versatile, highlighting improvements in both resistant and wild systems.

### 2.7. Heat Map

A Heat Map is a useful tool for comparing entities when a lot of data is available. It presents the general similarity or disparity between the studied elements in a simple proportional scheme of colors. It is applied in several fields of study, including molecular modeling. This approach can summarize all available data into one easier-to-interpret comparison.

**Figure 12:**
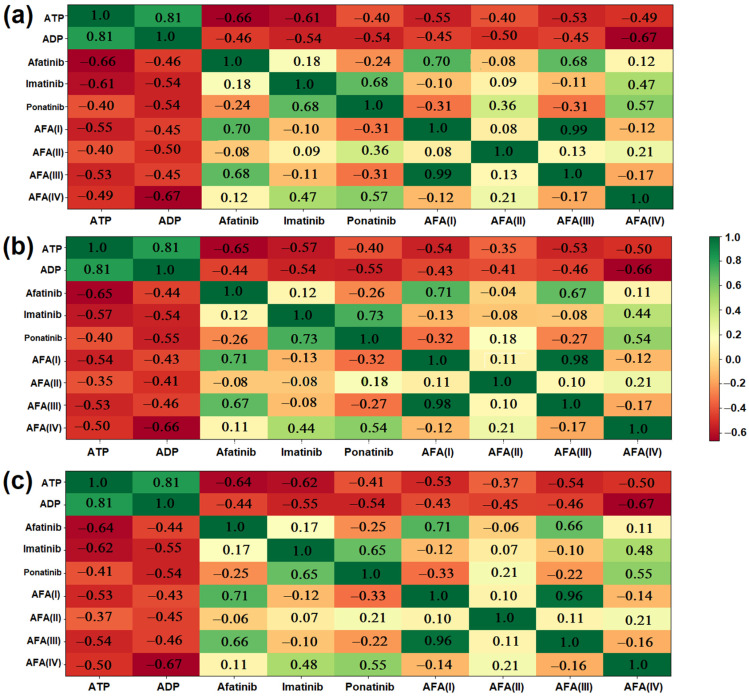
Heat maps showing the overall similarity between the derived molecules and the established inhibitors: (**a**) General similarity considering molecular descriptors; (**b**) General similarity considering molecular descriptors associated with the binding energy performance regarding the wild-type enzyme; (**c**) General similarity considering molecular descriptors associated with the binding energy performance regarding the mutated enzyme.

Several molecular descriptors, including ADMET and electronic properties, as well as docking results in both isoforms of the target protein and results related to the Fukui functions, were used as input values for a Python code that provided the Heat Maps presented in Figure 12.

The heat maps show a compilation of all presented data, providing a general comparison between the derived ligands and the established molecules. Figure 12a compares the molecules considering only their main molecular descriptors, electronic structure values, and Fukui functions. AFA(IV) presented 56.9% similarity with ponatinib, indicating that the modification successfully approaches the reference molecule. The same profile was also observed in the case of AFA(II), although to a lesser degree (35.7%).

While AFA(II) and AFA(IV) showed a good correlation with ponatinib, AFA(I) and AFA(III) remained close to afatinib (70.2% and 67.9%, respectively). This was expected since both these molecules were derived through the smallest modifications. They did not approach ponatinib or imatinib.

Figure 12b corroborates what is shown in graph (a), but it presents a clear division between the AFA(II) and ponatinib structures, with a similarity score of −32.1%. Since the second heat map considers the performance of the ligands regarding the wild-type enzyme, ponatinib is separated from AFA(II) because the latter appears to be a more versatile molecule.

The third heat map, shown in Figure 12c, considers the docking scores regarding the resistant enzyme. It also indicates AFA(IV) as a very close molecule to ponatinib (55.1%), as was suggested by the other maps and docking studies. AFA(II) again approached ponatinib (20.6%) but also imatinib to a lesser degree (6.7%). AFA(I) and AFA(III) remained close to afatinib (70.5% and 66.4%, respectively, corroborating that more significant modifications are necessary to reposition the original structure targeting Chronic Myeloid Leukemia.

## 3. Materials and Methods

Using bioisosterism and other strategies, we have evaluated four molecular structure entities derived from the afatinib molecule. This design was obtained through the GaussView software package version 4.1 [81]. The molecular structures of afatinib, imatinib, ponatinib, and the derived chemical entities are depicted in Figure 2. 

The AFA derivatives were designed as follows: AFA(I) has the relocation of the double bond in the carbon scaffold and substituting one fluorine atom with a chlorine atom in the aromatic ring. AFA(II) has the opening of the tetrahydrofuran ring, forming a new carbonyl group; AFA(III) has the relocation of the double bond in the carbon scaffold as well as a substitution of the oxygen atom linked with the five-membered ring by a sulfur atom. The AFA(IV) has the addition of a methylpiperazine ring to the molecule scaffold. The strategies involved in the modeling of AFA(I) and AFA(III) were classical monovalent and bivalent bioisosteric modifications aiming to provide softer centers (Cl and S) [55], which are more prone to polarization. AFA(II) should be more flexible and accept hydrogen bonds more easily with the presence of a carbonyl group, and AFA(IV) had the DFG-switch as a target since the methylpiperazine is present in the interaction with the Asp381 residue [56]. All modifications were done considering previous works [44,45,46,47,48].

The derived molecules were fully optimized to find the conformation related to the minimum global energy. For this purpose, the computational package Gaussian 16 was used [82]. Our approach involved the use of DFT with the hybrid functional B3LYP and the basis set 6-311+G(d,p) [83,84], following our recent studies [23,24,40,85]. The molecular structures were optimized in a vacuum, and the influence of the solvent was simulated through the implicit method of the Solvation Model Based on Electron Density (SMD), which considers water as the solvent [86]. This comprehensive approach ensures the accuracy and reliability of calculated data.

Molecular descriptors (molecular weight, number of aromatic heavy atoms, hydrogen bond acceptors, and donors, total polar surface area, LogP, general toxic effects) were obtained using the Osiris software package version 1.0 [70] and the SwissADME 14.9.29 [69] online tool to characterize the absorption, distribution, metabolism, excretion, and toxicity properties for all molecules analyzed in this study. The Electron Localization Function and Non-Covalent Interactions studies were performed using Multiwfn [87] with the purpose of modeling properly the electronic and molecular behavior of these molecules.

Docking studies of the classical TKIs, ponatinib, and imatinib and the derived AFA molecules applied to the human BCR-ABL enzyme were performed to evaluate the interactions between the classical and derived ligands and the biochemical targets. Two main docking studies were performed. The first study considered the mutated BCR-ABL form carrying the T315I mutation. The second docking study was taken using the wild-type variant of the enzyme. The wild form of the BCR-ABL is the *Mus musculus* enzyme in its wild type form and the DFG-out conformation (code 1OPJ) [88]. The mutated BCR-ABL protein crystallographic structure selected for our study is the *Homo sapiens* variant carrying the gatekeeper mutation and is in the DFG-out conformation (code 3QRJ) [78].

The docking studies were performed using the AutoDock Vina algorithm through the AutoDock Tools suite [77,89]. The configuration set of the docking study is in line with the literature [85,90,91,92,93]. AutoDock Vina searched for a maximum of 1000 solutions at an exhaustiveness level of 32. The 3QRJ protein grid parameters were set to width = 62, height = 64, and depth = 70, with the center point at X = 4.262, Y = -14.740, and Z = 28.439. Alternatively, the 1OPJ protein had its grid parameters set to width = 66, height = 62, and depth = 56, with the center point at X = 18.478, Y = 20.185, and Z = 54.223. 

All calculations were done with all protein residues in a rigid conformation while the ligand was allowed all degrees of freedom. The effectiveness of the rigid target/flexible ligand approach was discussed in the literature [94,95]. 

A redocking study was performed for both proteins to validate the docking protocol (Figure S4, supplementary material). In the case of the mutated enzyme, the value of the RMSD was 0.46 Å with a score of −13 kcal/mol for the wild form, and the deviation value was 0.23 Å with a score of −12.8 kcal/mol. The small values of the RMSD obtained through both redocking studies properly validate the docking protocol.

The complexes formed between the ligands and target proteins were further studied using the Multiwfn software package version 3.8 [87] to shine a light on the most relevant interactions. The NCI index (based on promolecular density) was analyzed to understand the interactions between the ligand and the target enzymes. The visualization of those interactions was rendered using the VMD software package version 1.93 [96].

Multiwfn was also used to perform additional studies of the small molecules, such as applying Fukui functions [51] to predict the electrophilic, nucleophilic, and radical reactive sites. These functions allowed for better detailing of the structures studied and provided a deeper understanding of the interactions between the ligands and the target protein when combined with the docking results. The central amide moiety, present in all molecules, was targeted for an Electron Localization Function [61] performed using Multiwfn to reveal the electronic profile of this functional group in the derived ligands when compared to the established ones. This ELF study was supplemented quantitatively by a Quantum theory of Atoms in Molecules analysis carried out through the AIMAll software package version 16.01.09 [97].

To validate the results observed in this study, data mining and statistical analysis were performed through a heat map graph that compared the main molecular descriptors (molecular weight, number of aromatic rings, total polar surface area, number of hydrogen bond donors and acceptors) and results obtained in electronic (chemical potential, softness, and electrophilicity index of all four aromatic nitrogen atoms) and docking studies (scores) to provide a general similarity index. This index was then used to compare and predict the established inhibitors by the derived molecules. The heat map analysis was done using the algorithm developed by our group using the Python language and respective libraries.

## 4. Conclusions

Four molecular structures were derived through modifications of the structure of afatinib. AFA(I) and AFA(III) did not present significant changes when compared to the original molecule These afatinib derivative molecules have demonstrated better interactions with both wild-type and mutated isoforms of the BRC-ABL enzyme, hinting at a promising class of TKI molecules. The modifications made to the original molecule have effectively eliminated predicted toxicity from afatinib. However, significant improvements were achieved through AFA(II) and AFA(IV) design. AFA(I) and AFA(III) conserved irritative properties, while AFA(II) and AFA(IV) provided a better toxicity profile according to the theoretical studies. Also, according to the modifications established in the afatinib molecule, these molecules were able to mimic ponatinib, although with less intense interactions when docked to mutant BCR-ABL enzyme. The group adjacent to the central amide appears to be heavily modulating the docking behavior, with large, positively charged groups (such as a methylpiperazine ring) possibly correlated to better scores. Additionally, electrophilic properties appear to be preferable, possibly conferring further affinity between the ligand and the target protein. The Arg386 residue appears to disrupt optimal docking conformation and possibly hinders inhibition. The affinity for this residue should be avoided when planning new molecules. On the other hand, residues Ile/Thr315, Met318, and Asp381 seem to be related to the best inhibitory conformation and must be the main targets of interaction. This analysis has provided deeper insights into the BCR-ABL system, allowing for a better understanding of the interaction of this enzyme and its ligands.

## Figures and Tables

**Table 1:** Electron densities at the Bond Critical Points (ρ) and Laplacian of the electron densities at the Bond Critical Points ($\nabla^{2}\rho$) of the amide function in all the studied molecules.

	ρ (CO)	$\nabla^{2}\rho$ (CO)	ρ (NH)	$\nabla^{2}\rho$
Afatinib	0.4017	−0.2541	0.3399	−1.745
Imatinib	0.3890	−0.3640	0.3396	−1.730
Ponatinib	0.3989	−0.3147	0.3421	−1.705
AFA(I)	0.2489	−0.2940	0.3384	−1.769
(AFA(II))	0.3756	−0.5108	0.3485	−1.806
(AFA(III))	0.3940	−0.3124	0.3370	−1.710
(AFA(IV))	0.3991	−0.3167	0.3412	−1.704

**Table 2:** Data provided by SwissADME and Osiris when predicting molecular descriptors of the studied structures and established ones.

	MW (Da)	LogP	# RT	# H-acc	# H-don	TPSA (Å^2^)	GA	Ro5	Bioavailability Score	Leadlikeness	Druglikeness
AFA(I)	503.40	2.92	9	4	3	87.22	High	1	0.56	2	1.77
AFA(II)	487.95	2.21	10	6	3	98.22	High	0	0.55	2	−0.53
AFA(III)	503.01	2.9	9	4	3	103.20	High	1	0.56	2	0.35
AFA(IV)	556.05	4.42	11	7	3	91.85	High	1	0.55	3	−0.91
Afatinib	486.95	3.64	9	5	3	88.61	High	0	0.55	3	−3.64
Imatinib	494.61	3.94	9	5	3	95.07	High	0	0.55	3	4.47
Ponatinib	535.58	3.86	8	7	2	65.77	High	1	0.55	3	2.10

MW: Molecular Weight; RT: Rotatable bonds; H-acc: H-bond acceptors; H-don: H-bond donors; TPSA: Total Polar Surface Area; GA: Gastrointestinal absorption; Ro5: Lipinski rules of five violations.

**Table 3:** Predicted mutagenic, tumorigenic, irritant, and reproductive effects of the derived molecules compared to that of the established ones.

Molecule	Mutagenicity	Tumorigenicity	Irritability	Reproductive Effects
AFA(I)	no effect	no effect	mild	no effect
AFA(II)	no effect	no effect	no effect	no effect
AFA(III)	no effect	no effect	mild	no effect
AFA(IV)	no effect	no effect	no effect	no effect
Afatinib	no effect	no effect	mild	no effect
Imatinib	no effect	no effect	no effect	no effect
Ponatinib	no effect	no effect	no effect	no effect

**Table 4:** Energy interaction values (kcal/mol) were obtained through docking studies for the derived ligands and the classical BCR-ABL TKI molecules.

Ligand	1OPJ	3QRJ	Experimental IC_50_ (nM)Wild-Resistant
AFA(I)	−8.7	−7.3	N.A.
AFA(II)	−8.9	−8.1	N.A.
AFA(III)	−8.3	−8.3	N.A.
AFA(IV)	−8.5	−9.0	N.A.
Afatinib	−8.8	−7.6	N.A.
Imatinib	−12.8	−8.9	260–6400 [74]
Ponatinib	−12.5	−11.8	0.37–2.00 [75]

## Data Availability

The datasets generated for this study can be found in the Supplementary Materials.

## Supplementary Materials

The following supporting information can be downloaded at: https://www.mdpi.com/article/10.3390/molecules29174254/s1, Figure S1. Fukui functions of ATP structure. Figure S2. 2D representation of the Vina docking conformations in the 1OPJ protein of classical inhibitors: (a) afatinib, (b) imatinib, (c) ponatinib. Figure S3. 2D representation of the Vina docking conformations in the 3QRJ protein of classical inhibitors: (a) afatinib, (b) imatinib, (c) ponatinib. Figure S4. Redocking study.
