# Supplementary material for: In Silico Molecular Modeling of Four New Afatinib Derived Molecules Targeting the Inhibition of the Mutated Form of BCR-ABL T315I
Source: Molecules. 2024 Sep 8;29(17):4254. doi: 10.3390/molecules29174254 (PMC11397288; doi:10.3390/molecules29174254)
Supplement: Supplementary file 1 [file molecules-29-04254-s001.zip › molecules-3078508-supplementary.pdf]

Analysis of electronic structure descriptors targeting molecular docking of afatinib derivatives

Kelvyn M.L. Rocha[1], Érica C.M. Nascimento[1,2], Rafael C. C. de Jesus[1], João B.L. Martins[1,2*]

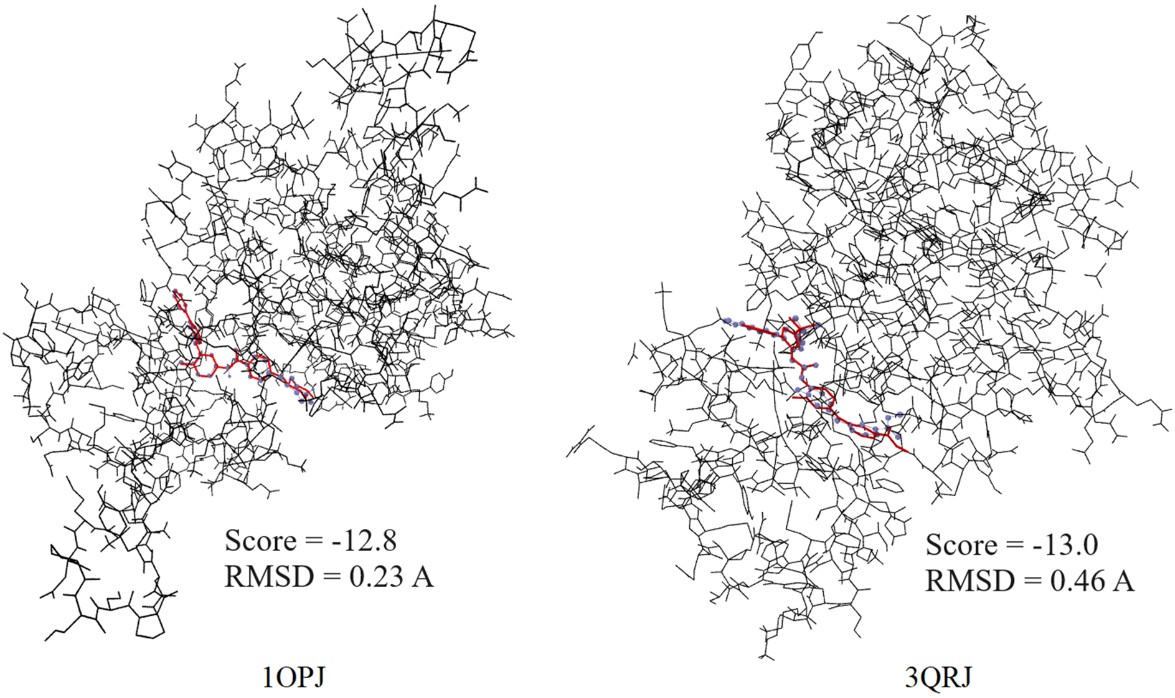

Figure S1. Redocking Study, scores in kcal/mol.

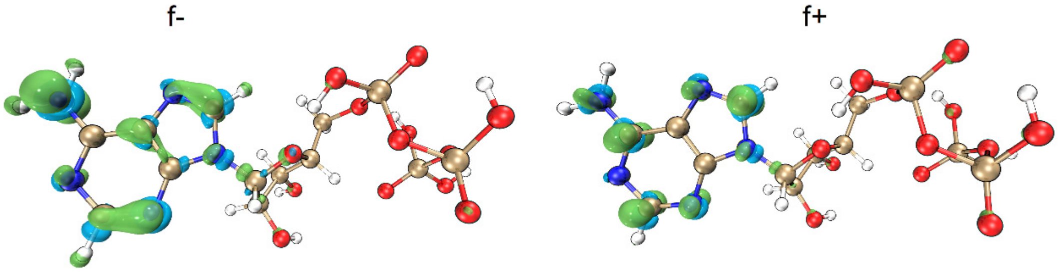

Figure S2. Fukui functions of ATP structure.

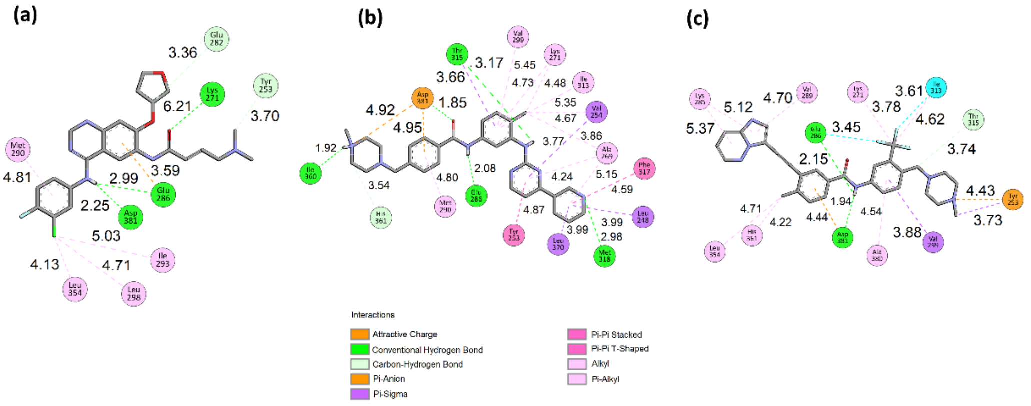

Figure S3. 2D representation of the Vina docking conformations in the 1OPJ protein of classical inhibitors: a) afatinib, b) imatinib, c) ponatinib.

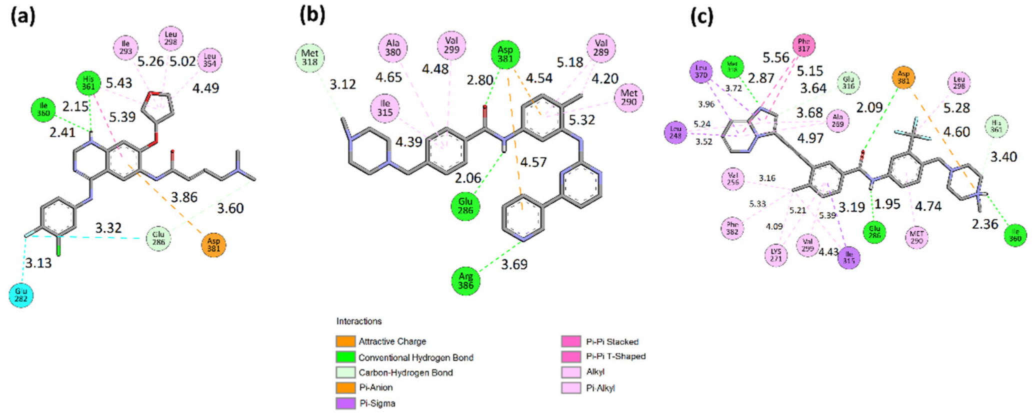

Figure S4. 2D representation of the Vina docking conformations in the 3QRJ protein of classical inhibitors: a) afatinib, b) imatinib, c) ponatinib.
